# Development of High-Tech Self-Compacting Concrete Mixtures Based on Nano-Modifiers of Various Types

**DOI:** 10.3390/ma15082739

**Published:** 2022-04-08

**Authors:** Sergey A. Stel’makh, Evgenii M. Shcherban’, Alexey Beskopylny, Levon R. Mailyan, Besarion Meskhi, Nikita Beskopylny, Yuriy Zherebtsov

**Affiliations:** 1 Department of Engineering Geology, Bases, and Foundations, Don State Technical University, 344003 Rostov-on-Don, Russia; sergej.stelmax@mail.ru (S.A.S.); au-geen@mail.ru (E.M.S.); 2 Department of Transport Systems, Faculty of Roads and Transport Systems, Don State Technical University, 344003 Rostov-on-Don, Russia; 3 Department of Roads, Don State Technical University, 344003 Rostov-on-Don, Russia; lrm@aaanet.ru; 4 Department of Life Safety and Environmental Protection, Faculty of Life Safety and Environmental Engineering, Don State Technical University, 344003 Rostov-on-Don, Russia; spu-02@donstu.ru; 5 Department Hardware and Software Engineering, Don State Technical University, 344003 Rostov-on-Don, Russia; beskna@yandex.ru; 6 Department of Technological Engineering and Expertise in the Construction Industry, Don State Technical University, 344003 Rostov-on-Don, Russia; yuri.zherebtsov@gmail.com

**Keywords:** self-compacting concrete, self-compacting concrete mixture, nano-modifier, nano-modified concrete, technological characteristics

## Abstract

Promising areas of concrete material science are maximum greening, reducing the carbon footprint, and, at the same time, solving the problems of increasing the cost of raw materials using industrial waste as modifiers for self-compacting concrete mixtures. This study aimed to review, investigate and test from the point of view of theory and practice the possibility of using various industrial types as a nano-modifier in self-compacting concrete with improved performance. The possibility of nano-modification of self-compacting concrete with a complex modifier based on industrial waste has been proved and substantiated theoretically and experimentally. The possibility of improving the technological properties of concrete mixtures using such nanomodifiers was confirmed. The recipe and technological parameters of the process were revealed and their influence on the characteristics of concrete mixes and concretes were expressed and determined. Experimental technological and mathematical dependencies between the characteristics of the technological process and raw materials and the characteristics of concrete mixtures and concretes were determined. The optimization of these parameters was carried out, a theoretical substantiation of the obtained results was proposed, and a quantitative picture was presented, expressed in the increment of the properties of self-compacting concrete mixtures using nano-modifiers from industrial waste concretes based on them. The mobility of the concrete mixture increased by 12%, and the fluidity of the mixture increased by 83%. In relation to the control composition, the concrete strength increased by 19%, and the water resistance of concrete increased by 22%. The ultimate strains decreased by 14%, and elastic modulus increased by 11%.

## 1. Introduction

The growing requirements for the quality of building materials, products, and structures [1,2,3], the emergence of unique buildings and structures, high and long-span, set the primary areas of scientific tasks in building materials, innovative technologies, production methods, compositions, technological, and highly functional concretes of a new generation. In this regard, it is conditionally possible to divide the promising areas of concrete science into two aspects. The first is the achievement of high-performance characteristics of the resulting concrete for the construction of unique buildings and structures of increased responsibility from them. The second is the maximum manufacturability and ergonomics of the construction processes themselves, which are primarily due to the possibility of obtaining these reinforced concrete products, structures, buildings, and structures with acceptable quality under challenging conditions and within a reasonable time.

Thus, this article focuses attention on high-tech processes for constructing reinforced concrete and concrete buildings and structures of a new type. As is known, in current conditions all over the world, the main requirements are not only high quality and speed of construction of buildings and structures, but also the maximum environmental friendliness and efficiency of such construction in the face of rising prices for energy, raw materials, machinery and equipment, and increased requirements for the wages of workers. Thus, one of the main directions is also greening and achieving maximum efficiency of construction processes in the construction of reinforced concrete buildings and structures. In this regard, one of the promising areas is the development of the most technologically advanced, highly functional concrete mixtures of a new generation, which allows achieving savings using a minimum amount of means for transportation, laying, compaction, care of hardening concrete, the possibility of the fastest construction of buildings and structures with the most attentive ensuring proper quality. Self-compacting concrete mixtures are mixtures of a new generation. They allow bypassing such problems of current construction as the high-density of reinforcement and the impossibility of manual or machine compaction of the concrete mixture in hard-to-reach concrete structures, minimize costs for all stages of the life cycle of the construction process, and ensure the excellent quality of the construction process as soon as possible in the construction of reinforced concrete buildings and structures. From this point of view, concerning improving the quality and manufacturability of self-compacting concrete, many works are known.

At the same time, as mentioned above, a critical condition is maximum greening, reducing the carbon footprint, using the possibility of maximum greening of the environment, reducing the environmental burden, and solving the problems of increasing the cost of raw materials. In this regard, the search for technological, constructive, recipe approaches, the use of several types of waste, industrial and agricultural and other wastes of economic entities and humans as modifiers of self-compacting concrete mixtures, are also seen as promising areas.

Superplasticizers can produce self-compacting concretes (SCC) containing various particulate materials such as Portland cement, mineral additives, fillers, and fine and exceptionally fine sand. The addition of a superplasticizer makes it possible to preserve the rheological properties of the concrete mixture for a time sufficient for the transportation and molding of products and structures [4]. The influence of various dosages and types of superplasticizers on the workability of self-compacting concrete mixtures and the parameters of the hardened SCC were considered in [5,6,7]. For example, various types of superplasticizers are based on polycarboxylates, lignosulfonic acid, melamine-formaldehyde sulfonic acid naphthalene-formaldehyde sulfate acid, had different effects on the flow and loss of fluidity of concrete mixtures [6]. At the same time, the mechanism of action of the additive is also essential, as well as its exact dosage for each specific case of the use of self-compacting concrete [7].

The use of finely dispersed mineral additives makes it possible to improve the technological characteristics of self-compacting concrete mixtures and the physical and mechanical properties of concrete. At the same time, the most significant effect in the mentioned improvement can be achieved when using rationally selected combinations and dosages of mineral additives together with a superplasticizer, optimizing the granulometric composition of aggregates, which makes it possible to obtain high-strength concrete as a result [8,9,10]. The works [11,12,13,14,15,16,17,18,19,20,21,22,23,24,25,26,27,28] describe the mechanisms of action of various mineral additives; for example, fly ash from thermal power plants, ground blast-furnace granulated slag, metakaolin, micro-silica, and present various dosages of compositions of self-compacting concrete mixtures both with one type of mineral additive and with several. Improvements in the characteristics of self-compacting concrete mixes and concrete obtained in [11,12,13,14,15,16,17,18,19,20,21,22,23,24,25,26,27,28] from finely dispersed mineral additives confirm the effectiveness of their use and the need for accurate selection of the concrete composition based on them. Furthermore, an essential role in forming the structure of concrete and interparticle interactions is played using finely dispersed mineral material-micro- (nano-) silica. Due to its properties and granulometry, it creates a dense three-dimensional framework of chains and aggregates with numerous coagulation contacts with other larger particles. Therefore, the use of silica fume and other mineral additives listed above enhances the effect and further improves the technological characteristics of self-compacting concrete mixtures and the physical, mechanical properties [29,30,31,32,33].

The structure-forming role of mineral additives is to reduce the induction period of structure formation due to the adsorption of hydrolysis products and to increase the time to reach supersaturation of the liquid phase. Thus, the mineral additive activates the processes of hydration of the binder and promotes an increase in the volume and degree of crystallinity of the formed hydrates, which contributes to the compaction of the structure at the point of contact with the additive with a significant increase in the microhardness of hydrate aggregates [34].

The joint inclusion of a superplasticizer and a mineral additive into a self-compacting concrete mix increases the effect compared with the use of only one of these types of additives. In the example of cement stone, the differences in the relative value of the modulus of elasticity are presented when using only a superplasticizer (an increase of up to 10%) and when using a superplasticizer with a mineral additive (an increase of up to 20%) [27,28].

Thus, the use of complex additives (chemical and mineral) makes it possible to reduce the consumption of cement, maintain the workability of concrete mixtures for a long time, increase the density of the structure, and, as a result, the strength, durability, and resistance of concrete under aggressive operating conditions [10].

In [35], the authors studied the “effect of granulated blast-furnace slag, and two types of superplasticizers on the properties of self-compacting concrete. The results showed that concrete mixtures with a polycarboxylate-based superplasticizer provided more excellent workability and higher compressive strength for all ages than those with a naphthalenesulfonate-based superplasticizer.” In [29], the authors conducted experimental studies to assess the effect of micro-silica and a viscosity modifier on the mechanical and rheological properties of self-compacting concrete.

One of the most “focused on the maximum use of industrial waste as modifying additives for self-compacting concrete” are works [10,36,37,38]. The authors of these works carried out theoretical and experimental studies, which are of particular interest to us. In [10], the influence of a polyfunctional modifier on the properties of concrete mixtures and concrete was studied. For this, the rheological properties of cement pastes used in the compositions of SCC were determined. It was established that a polyfunctional modifier in the form of a concentrated suspension of microsilica in a solution of sodium hydroxide and a superplasticizer based on polymethylene naphthalenesulfonate reduces plastic viscosity and slightly reduces the yield strength. The regularities of influence of rheological properties of cement paste with organomineral modifier on the properties of self-compacting concrete mix was established. As a replacement for cement in the amount of 35–45%, ground blast furnace granulated slag was used. It was also established that the multicomponent modifier improves the properties (increase in slump flow and slump retention) and hardened SCC [36]. This study, in many ways, sets itself the task of developing the existing ideas and achievements of the authors [10,36,37,38].

Having reviewed and analyzed the previously achieved results and technological methods obtained by other authors [1,2,3,4,5,6,7,8,9,10,11,12,13,14,15,16,17,18,19,20,21,22,23,24,25,26,27,28,29,30,31,32,33,34,35,36,37,38,39,40,41,42,43,44,45], it can be noted that the use of various industrial wastes as modifier additives have already been announced as promising areas. However, it should be emphasized that the use of such wastes in a processed form, crushed to the state of nanoparticles, thereby molds them into the form of nano-modifiers. Nanomodification of building materials, particularly concretes and self-compacting concretes, is one of the main fundamental areas of building materials science. Summing up the results of the literature review and the conclusion made, it can be formulated a working hypothesis of the study, define the study’s purpose and objectives, and formulate the proposed scientific novelty and practical significance of the study.

## 2. Materials and Methods

### 2.1. Hypothesis, Aim, and Objectives of the Study

The working hypothesis of the study is the possibility of improving the manufacturability of self-compacting concrete mixtures using nano-modifying additives from several types of waste, thereby making it possible to achieve an increase in the manufacturability, efficiency, and environmental friendliness of the construction process.

The study is aimed to review, investigate, and evaluate from the point of view of theory and practice the possibility of using various types of industrial and agricultural waste as nano-modifiers in self-compacting concrete with improved characteristics.

The research objectives comprise:

- formulation of the problem based on data on the modification of concrete mixtures with a reorientation of the study in the direction of nano-modification with the waste of this type;
- conducting approbation studies with a detailed study of the initial components selected based on the most common wastes that accumulate in large quantities and pose the greatest potential threat to the environment;
- after selecting, determining, and establishing the initial characteristics of the base raw material-determining the working compositions for optimizing and testing the hypotheses put forward, conducting large-scale experimental studies with fixing the technological characteristics of self-compacting concrete mixtures as criteria and checking them to verify the resulting characteristics of highly functional concrete from such mixtures;
- summarizing the results of the study, determining the technological properties of concrete mixes and the performance characteristics of concrete, putting forward subsequent scientific hypotheses, and developing proposals for applied use in building materials science and technology of building processes, determining the goals and objectives of subsequent research, as well as proposals for industrial approbation of the results obtained after their detailed comparison with the results of other authors and understanding of the scientific novelty and place of the research in construction science and practice.

**Figure 1:**
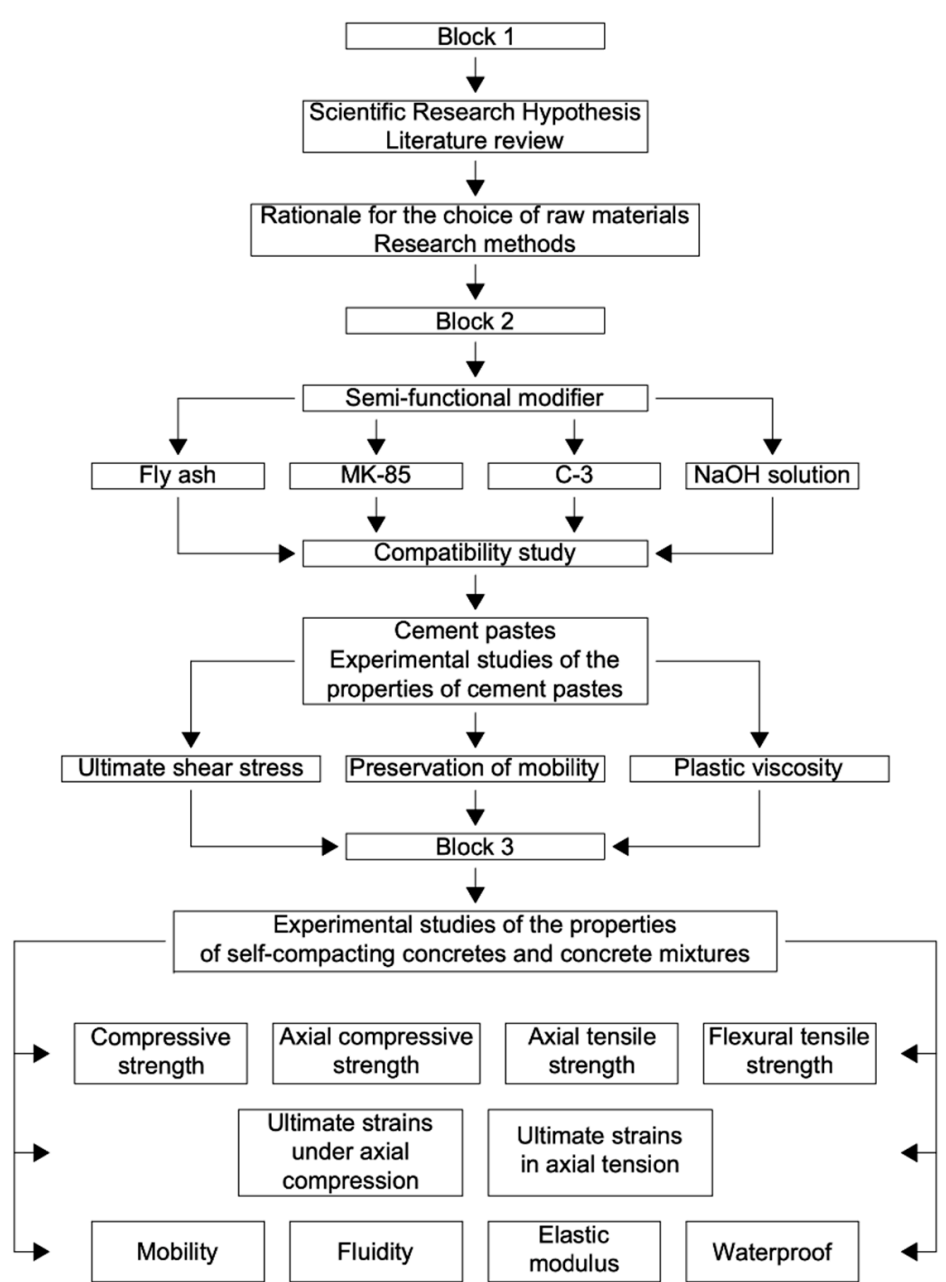
The research program.

The program study is presented in Figure 1.

### 2.2. Materials

Considering the complexity of the study and its division into the study of the technological parameters of the concrete mixture, and the verification of the results achieved by the characteristics of the obtained concrete, a complete list of the materials and research methods used are presented.

When conducting experiments for the preparation of cement pastes and self-compacting concrete mixtures, Portland cement PC 500-D0-N produced by Holcim (Rus) LLC (Volsk, Russia) was used as a binder. The chemical and mineralogical composition of Portland cement clinker is presented in Table 1 and Table 2, and the physical and mechanical properties of cement are presented in Table 3.

As mineral additives, the following are accepted: fly ash from the Novocherkassk State District Power Plant (Novocherkassk, Russia) and micro-silica grade MS-85 produced by ZIPo LLC (Lipetsk, Russia). The chemical composition and physical and mechanical characteristics of fly ash are given in Table 4; the chemical composition of micro-silica is presented in Table 5.

Granite crushed stone produced by Pavlovsknerud JSC (Pavlovsk, Russia) was used as a coarse aggregate, and quartz sand (*M*_f_ = 2.2) produced by Arkhipovsky Quarry JSC (Arkhipovskoe village, Russia) and quartz sand (*M*_f_ = 1.2) produced by Quartz Sands LLC (Semenov, Russia). Physical and mechanical properties of aggregates are presented in Table 6.

The following are accepted as chemical additives: sodium hydroxide produced by OOO “KHIMEKS” (Moscow, Russia); superplasticizer C-3 manufactured by Component LLC (Vladimir, Russia). The qualitative characteristics of the additives used are presented in Table 7 and Table 8.

### 2.3. Methods

The research methods can be conditionally divided into groups:

- analytical methods and literature data study;
- phenomenological methods for the selection and determination of raw materials, compositions, and the setting of allowable limits and ranges;
- experimental studies to verify the selected recipe and technological solutions;
- mathematical methods of substantiation and optimization of the developed proposals.

Experimental studies were carried out using standardized and unique methods, including:

- determination of the characteristics of the initial raw materials by standardized methods for binder and aggregates and research methods of laser granulometry for nano-modifying additives;
- determination of the technological characteristics of concrete mixes by standardized methods with verification of the results by the author’s method;
- determination of the characteristics of the resulting concrete by standardized methods [46,47,48,49,50,51,52,53,54,55,56].

The determination of the granulometric composition of powdered raw materials (fly ash and micro-silica) was carried out using the method of laser granulometry. A Microsizer 201C laser particle analyzer (OOO VA Install, St. Petersburg, Russia) was used for granulometric analysis [52].

X-ray phase analysis (XPA) of mineral additives was carried out on an X-ray diffractometer HZG-4C (Freiberger Prazisionmechanik, Germany).

The rheological properties of cement pastes (plastic viscosity, ultimate shear stress) were studied using a B085-21 rotational viscometer (OOO RNPO RusPribor, St. Petersburg, Russia). The results were processed according to the Bingham rheological model [10]:

(1) $$\tau =\tau_{0}+\mu \gamma$$

where $\tau_{0}$ is ultimate shear stress, (Pa); μ is plastic viscosity, (Pa×s); γ is shear rate gradient (s^−1^).

**Figure 2:**
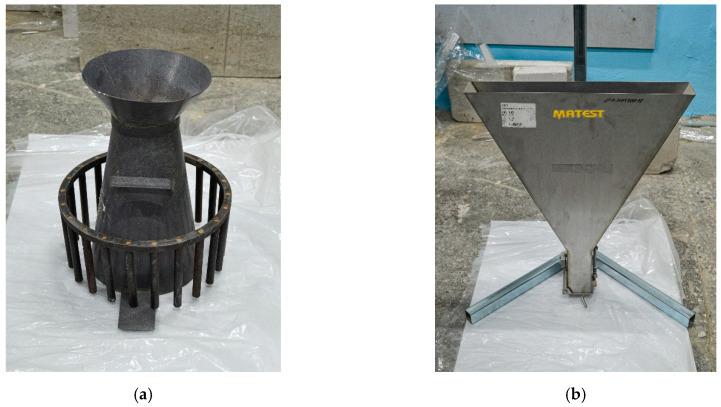
Devices for monitoring the rheological characteristics of SCC mixtures: (**a**) a cone for determining mobility; (**b**) V-shaped flow funnel.

The workability of a self-compacting concrete mixture is characterized by its ability to slump-flow and fill a given shape under the action of its mass or external forces while maintaining uniformity and solidity. Indicators of the technological properties of self-compacting concrete mixtures are such characteristics as mobility, expressed by the diameter of the flow of a standard cone and fluidity, expressed by the flow time of the concrete mixture from a V-shaped funnel. The mobility of the concrete mixture was estimated by the diameter of the flow in millimeters using a standard cone (RNPO RusPribor LLC, St. Petersburg, Russia), the appearance of the cone is shown in Figure 2a. The flow time of the self-compacting concrete mixture was determined using a V-shaped funnel Matest C171 (Treviolo, Italy). This funnel is made of stainless steel and mounted on a tripod. The inside has a smooth surface, the edges of the upper hole are reinforced with stiffeners, and the bottom hole is sealed (Figure 2b).

To evaluate and verify the results of standard methods, taking into account the specifics of the resulting mixtures, additional tests were carried out using a non-standard method, according to [57].

**Figure 3:**
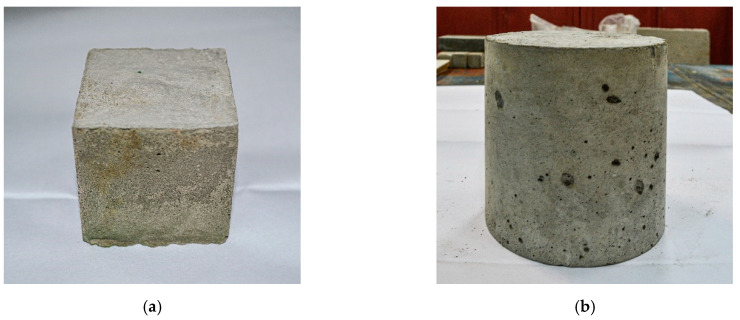
Concrete samples: (**a**) cubes; (**b**) cylinders.

Compression tests were carried out on 100 mm × 100 mm × 100 mm sample cubes (Figure 3a) of six pieces for each SCC composition. In addition, axial and bending tensile strength tests were carried out on 100 mm × 100 mm × 400 mm prism specimens of three pieces for each composition and type of SCC strength according to the requirements GOST 10180 “Concretes. Methods for strength determination using reference specimens” [58].

Determination of the elastic modulus and axial compressive strength (three prism samples 100 mm × 100 mm × 400 mm for each SCC composition) was carried out following the requirements of GOST 24452-80 “Concretes. Methods of prismatic, compressive strength, modulus of elasticity and Poisson’s ratio determination” [59].

Measurements of the strains of the concrete samples were carried out with the chain of strain gauges with a base of 50 mm and dial indicators with a division value of 0.001 mm on the experimental prisms. In addition, tests of experimental prisms for axial compression and axial tension were carried out at a constant strain rate to obtain the strength and strain characteristics of concrete and its complete strain diagrams “σ-ε” with descending branches.

The water-resistance of SUB samples was determined by the “wet spot” method following the requirements of GOST 12730.5 “Concretes. Methods for determination of water tightness” [60] on cylindrical specimens with a diameter of 150 mm and a height of 150 mm (Figure 3b). Six prepared samples of each composition were stored in a standard hardening chamber at a temperature of 20 ± 2 °C and relative air humidity of at least 95%.

**Figure 4:**
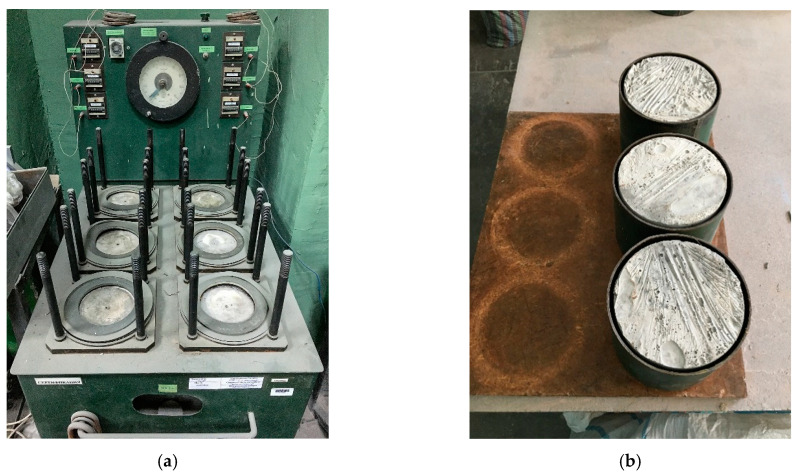
Concrete water resistance test: (**a**) UVF-6 installation; (**b**) concrete specimens in test cases.

The water pressure was increased stepwise with 0.2 MPa for 1–5 min and maintained at each step for 16 h (Figure 4). The water-resistance of each sample was evaluated by the maximum pressure of water at which no leakage through the sample was observed in the form of a wet spot on the end surface of the sample opposite to its surface through which water was supplied under pressure. The water-resistance of a series of concrete samples was evaluated by the maximum water pressure at which no seepage was observed on four of the six samples.

All manufactured prototypes hardened under normal conditions at a temperature of 20 ± 2 °C for 28 days.

The study used mathematical planning of the experiment, which aims to increase the efficiency of experiments.

In particular, the Box–Wilson method was applied-orthogonal planning of the optimal experiment. The essence of the chosen method is as follows:

In the first stage, for some local area of existence of the object under study, the regularities of its behavior are determined by the method of planning the experiment, and as a result of their analysis, the direction to the optimum is determined, in which the parameters should be changed. Next, another experiment is carried out in a new area of the object’s existence, and so on, until optimal conditions are reached.

Mathematical planning of an experiment is a formalized procedure for choosing the number and conditions for conducting experiments that are necessary and sufficient to solve a problem with the required accuracy. This procedure allows for the following:

(a) minimizing the number of experiments;
(b) changing the parameters that affect the state of the object according to a certain law;
(c) using a mathematical method that formalizes the actions of the experimenter in data processing and obtain a mathematical model of the research object;
(d) use the logical apparatus when making decisions based on the analysis of the resulting model.

Following the recommendations [10,36,37,38], it is possible to correctly assess the effectiveness of nano-modification of self-compacting concrete mixtures by analyzing the following experimental operations:

- revealing the influence of the type of dispersion medium on the efficiency of dispersion of the nano-modifier;
- to determine and evaluate the rheological properties of cement pastes with a complex nano-modifier;
- based on the data obtained, optimize the compositions according to the criteria of strength and deformability of concrete and the mobility of concrete mixtures.

## 3. Results

### 3.1. Determination of the Characteristics of Raw Components

**Figure 5:**
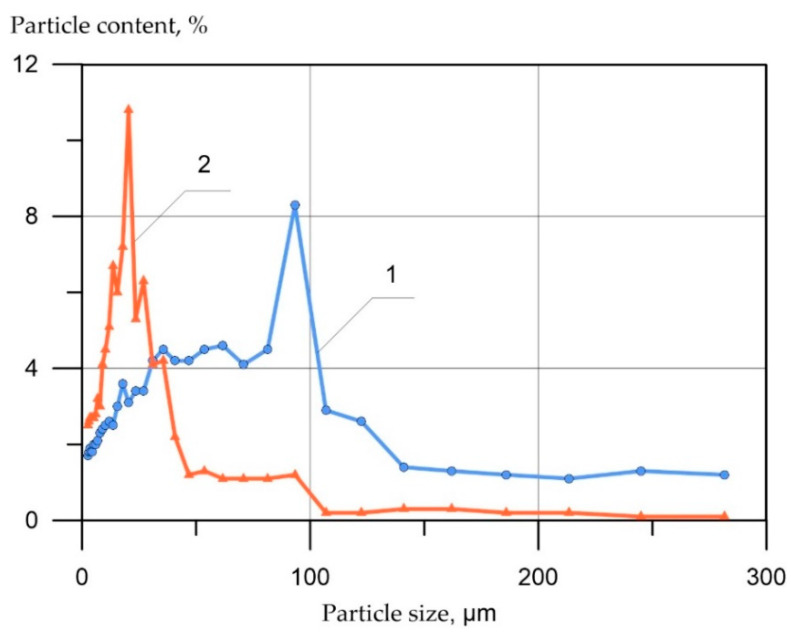
Particle size distribution determined on the laser analyzer “Microsizer 201C”: 1—fly-ash; 2—micro-silica MS-85.

The granulometric characteristics of the mineral additives are shown below in Figure 5.

Following the data shown in Figure 5, it can be seen that the main size distribution range of the studied fly ash particles is in the range of 2–120 µm, and the main peak falls at 90 µm. On the other hand, as for micro-silica particles, the predominant part of the grains (more than 80%) is located in the range from 2 to 40 µm, and the main peak falls at 20 µm.

**Figure 6:**
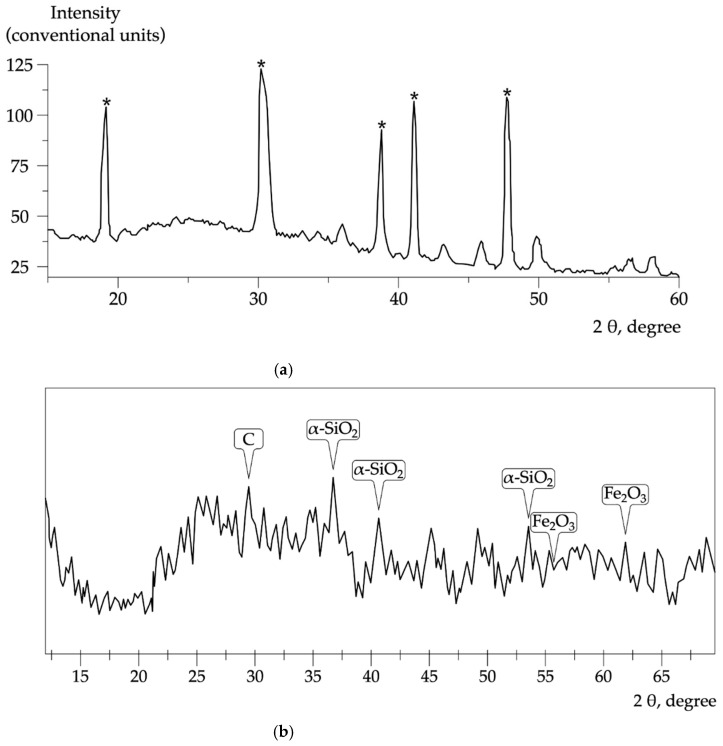
Diffractogram of samples of (**a**) fly ash (*—mullite phase); (**b**) micro-silica.

X-ray phase analysis of fly ash and micro-silica particles is shown in Figure 6.

X-ray diffraction analysis shows that the fly ash microspheres (Figure 6a) are a mixture of two phases: Al_6_Si_2_O_13_ mullite and an X-ray amorphous phase referred to as a glass, while the main phase is the mullite phase. Micro-silica (Figure 6b) is represented by amorphous silica, minor impurities of iron, carbonaceous substances, and crystalline α-quartz.

### 3.2. Influence of the Type of Dispersion Medium on the Efficiency of Micro-Silica Dispersion

Following the recommendations [10,36,37,38], a comparative analysis of three variants of dispersion media was carried out which included:

- tap water;
- sodium hydroxide solution (concentration 1.5% in terms of Na_2_O);
- solution of sodium hydroxide and superplasticizer (concentration 2.5%).

The preparation of an aqueous suspension of silica fume was carried out in a laboratory planetary ball mill “Activator-4M” for 8–12 min.

After the dispersion process, the predominant part of the micro-silica particles (more than 80%) is in the range of 2 to 35 µm, and the main peak falls at 10 µm.

**Figure 7:**
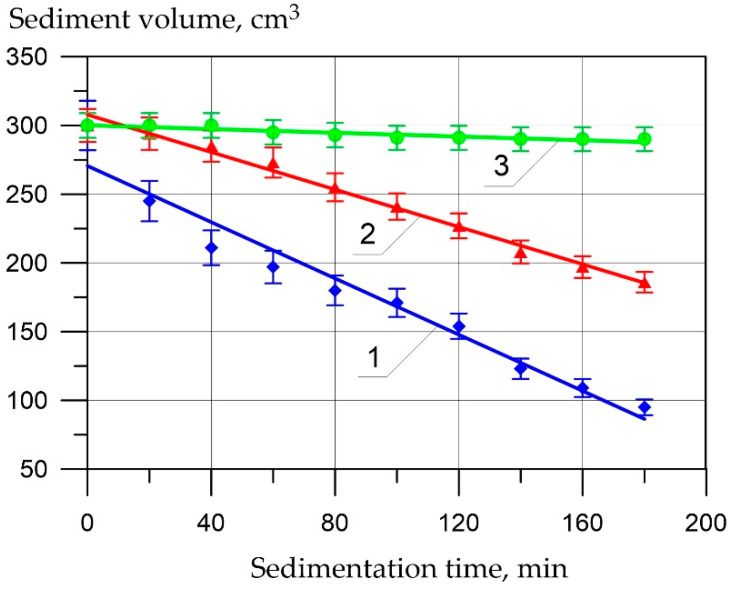
The kinetics of sedimentation of micro-silica suspension dispersed in the medium: 1—tap water; 2—sodium hydroxide solution; 3—sodium hydroxide solution + superplasticizer.

The dispersing ability of liquids was assessed by the kinetics of sedimentation of aqueous suspensions of silica fume (Figure 7), the concentration of the MS-85 additive in the aqueous suspension was 35%.

The authors of works [10,36,37,38] provide a theoretical justification that the highest particle settling rate is observed when tap water is used as a liquid medium. In a sodium hydroxide solution, the precipitation rate decreases, indicating the resulting dispersion’s finer granulometric composition. This is due to the fact that in a highly alkaline medium, along with the dispersion of micro-silica aggregates, it also dissolves.

In the presence of alkali, silicon dioxide goes into solution in the form of a silicate ion, followed by interaction with water to form soluble mono silicic acid. When the amount of alkali is small, mono silicic acid can polymerize to form stable colloidal particles. In the soluble sodium silicate formation process, an additional dispersion of amorphous silica occurs with a transition to a highly concentrated state-a lyosol nano dispersed system. In addition, the degree of micro-silica grinding is exclusively influenced by the self-heating temperature of the suspension during grinding, which is achieved mainly due to the work of friction.

When micro-silica is dispersed in the medium of sodium hydroxide and superplasticizer solutions, a colloid system stabilized by a surfactant is formed, which retains sedimentation stability for more than 10 days [10,36,37,38].

Thus, a solution of sodium hydroxide and a superplasticizer (concentration 2.5%) was chosen as the dispersion medium.

### 3.3. Rheological Properties of Cement Pastes with a Complex Nano-Modifier

**Figure 8:**
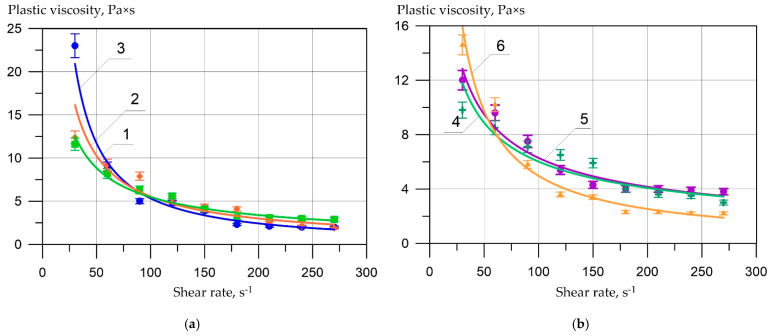
The plastic viscosity of cement pastes depends on shear rate: (**a**) 1—C1; 2—P1; 3—P2; (**b**) 4—P3; 5—P4; 6—P5.

Further, following the developed program, it was necessary to evaluate the rheological properties of cement pastes with different content of complex nano-modifiers (Table 9 and Figure 8). The rheological characteristics of cement pastes were determined as an average of three samples of each composition and are presented in Table 9.

During experiments on the selected initial formulation and technological parameters, the data [10,36,37,38] confirmed that the maximum value of structural viscosity at a minimum shear rate gradient of 30 s the value of normal density. When a superplasticizer is added, the water demand of the cement paste (P1) decreases to W/W = 0.20. In this case, the value of structural viscosity decreases by almost two times, and the equilibrium plastic viscosity at a shear rate of 240–270 s-1 is the smallest among all the studied compositions of cement pastes. At the same time, the addition of sodium hydroxide has a weak plasticizing effect, reducing the water requirement of the fly ash paste (P2) to W/W = 0.29.

To summarize, it can be noted that the partial replacement of Portland cement with fly ash in 40% slightly increases the water demand and plastic viscosity of the cement paste (P3) as established [10,36,37,38]. At the same time, a positive effect of a complex additive in the form of a superplasticizer C-3 + NaOH (P4) was noted. On the other hand, the relatively low content of micro-silica increases both the water demand and the plastic viscosity of the cement paste (P5). This effect should have a positive impact on the SCC resistance to delamination. Also, as in [10,36,37,38], we have not established a significant effect of the complex nano-modifier on the indicators of the ultimate shear stress of the cement paste.

### 3.4. Optimization of the Composition of Complex Nano-Modifiers According to the Criterion of Concrete Strength and Mobility of Concrete Mixtures

To optimize the composition of concrete (modifier) in terms of the consumption of mineral and chemical additives, studies were carried out using the method of orthogonal compositional planning of the 2nd order of Box–Wilson, which, under specific optimal parameters, allows achieving the maximum strength of concrete at the age of 28 days while ensuring the required mobility of concrete mixtures. Thus, the compressive strength (*R*_b,cub_), mobility (*d*_sl_), and flow (*T*_fl_) parameters served as optimization parameters. The basic composition of concrete was adopted for testing, determined according to the method [61]. As a result of calculations, the parameters of the composition of the concrete mix are shown in Table 10.

The values of the variation factors, their physical meaning, and variation levels are given in Table 11, the planning matrix-in Table 12. The compressive strength of concrete was determined on sample cubes with an edge of 100 mm at 28 days of normal hardening. The results of the experiments are shown in Table 13.

Having calculated the coefficients of orthogonal central planning of the 2nd order, the following regression Equations (2)–(4) were obtained:

(2) $$\begin{array}{ll} R_{\left(b,cub\right)}= & 50.618-1.698\;\cdot \;N-6.941\;\cdot \;F+0.974\;\cdot \;C-0.283\;\cdot \;M+0.785\;\cdot \;N\;\cdot \;F+1.284\;\cdot \;N\;\cdot \;C\\ & -0.586\cdot N\cdot M+0.150\cdot F\cdot M-0.349\;\cdot \;C\;\cdot \;M-3.828\;\cdot \;N^{2}-3.370\;\cdot \;F^{2}-1.746\;\cdot \;C^{2}-2.229\;\cdot \;M^{2} \end{array}$$

(3) $$\begin{array}{ll} d_{spr}= & 546.592-17.061\cdot N-1.248\cdot F+64.500\cdot C-7.074\cdot M-1.870\cdot N\cdot F+16.831\cdot N\cdot C\\ & +6.857\cdot N\cdot M\;+3.117\cdot F\cdot M-5.610\cdot C\cdot M+9.713\cdot N^{2}-18.395\cdot F^{2}-1.040\cdot C^{2}-18.395\cdot M^{2} \end{array}$$

(4) $$\begin{array}{ll} T_{fl}= & 22.653+0.375\cdot N+0.458\cdot F+3.038\cdot C+1.290\cdot M+0.436\cdot N\cdot F+0.187\cdot N\cdot C+0.062\cdot N\cdot M\\ & -0.187\cdot F\cdot M+2.307\cdot C\cdot M-0.326\cdot N^{2}+0.015\cdot F^{2}-1.348\cdot C^{2}-2.588\cdot M^{2} \end{array}$$

The significance of the regression equation coefficients was checked using the Student’s test, and the adequacy of the regression equations to the experimental data was checked using the Fisher criterion.

After verification, insignificant coefficients were discarded, resulting in Equations (5)–(7):

(5) $$R_{\left(b,cub\right)}=50.618-6.941\cdot F-3.828\cdot N^{2}-3.370\cdot F^{2}$$

(6) $$d_{spr}=546.59+64.50\cdot \;C$$

(7) $$T_{fl}=22.653+3.038\cdot C+2.307\cdot C\cdot M-2.588\;\cdot M^{2}$$

**Figure 9:**
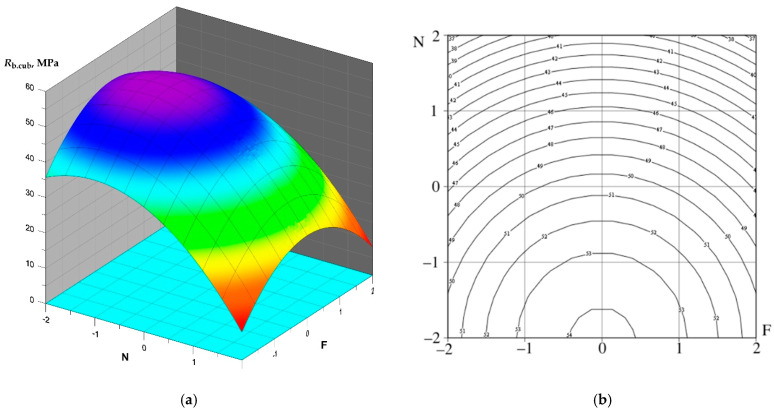
Dependence of compressive strength on NaOH concentration and fly ash content: (**a**) plot surface; (**b**) surface contours.

**Figure 10:**
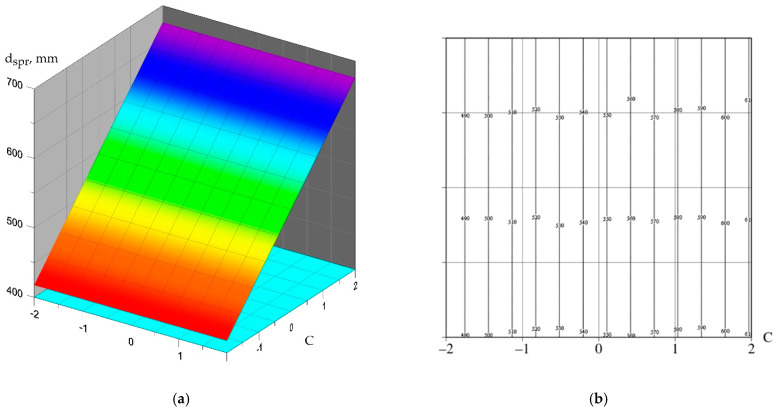
The dependence of the diameter of the flow of the concrete mixture on the content of C-3: (**a**) dependence surface; (**b**) surface contours.

**Figure 11:**
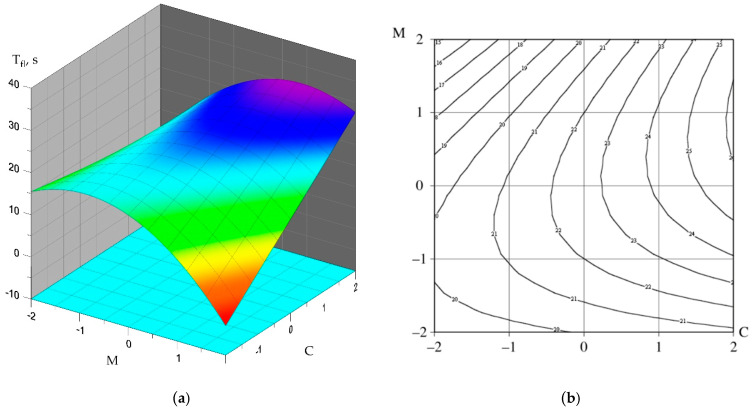
Dependence of the fluidity of the concrete mix on the content of C-3 and silica fume: (**a**) the dependence surface; (**b**) surface contours.

Graphical interpretation of the obtained polynomial dependencies is shown in Figure 9, Figure 10 and Figure 11.

An analysis of the regression equations shows that the factors X_1_ and X_2_ have the greatest influence on the compressive strength of the SCC. The optimal dosage of fly ash introduced to replace part of the cement is a dosage of 40% by weight of cement. As for the dosage of NaOH, the optimal dosage is 0.8% by weight of cement.

It should be noted that for compositions No. 3, 4, 7, 8, 11, 12, 15, 16, where the content of fly ash in the SCC composition is 50%, lower values of compressive strength are observed in comparison with other compositions and vary from 28.9 MPa and up to 33.6 MPa; the minimum strength value was recorded for composition No. 22 with a fly ash dosage of 55% and is 27.8 MPa.

The X3 factor has a significant impact on mobility, increasing the dosage of the C-3 plasticizer from 1.1% to 1.8% increases the mobility of the self-compacting concrete mix. The maximum value of the mixture flow diameter was recorded for composition No. 22, where the C-3 dosage was 1.8% of the cement weight.

As for such a parameter as the flowability of a self-compacting concrete mix, the factors X_3_ (C-3 content by cement mass, %) and X_4_ (MS-85 content by cement mass, %) have the greatest influence on this indicator. The highest values of fluidity were recorded for compositions No. 13, 14, 16, 20, and 22 and amounted to 25–26 s. The lowest values of fluidity are observed in compositions No. 1, 2, 3, 4, 5, 6, 7, 8, 9, 10, 11, 23 and vary from 13 to 17 s. An increase in the dosages of superplasticizer and micro-silica additives contributes to an increase in the flow time of the concrete mixture, which can be directly related to an increase in the viscosity of the mixture itself.

### 3.5. Rheological and Physical-Mechanical Properties of Self-Compacting Concrete with a Complex Nano-Modifier

Table 14 shows the compositions of the self-compacting concrete mixture of the base composition with a complex nano-modifier.

Comparative tests of concretes and concrete mixtures of the control composition (CC) and with a complex nano-modifier (CNM) are presented in Table 15.

Comparative tests of the control composition of concrete and nano-modified one show that the alkaline activator in the composition of the complex nano-modifier allows partial replacement (40%) of Portland cement with fly ash while increasing the compressive strength of concrete by 17%, the axial compressive strength by 10%, the strength for axial tension by 19%, and the tensile strength in bending by 11%. Ultimate strains under axial compression and tension decreased by 14% and 12%, respectively. The modulus of elasticity increased by 11%. As for water resistance, its value increased by 22%.

As can be seen in Table 13, CNM concrete samples are characterized by higher strength in the early stages of hardening (by 18% on day 2 and by 14% on day 7) compared to CC samples.

Higher indications of the early strength of the CNM composition can be theoretically justified in accordance with [10,36,37,38] because primary aluminate phases begin to form more intensively, providing concrete strength indicators at an early age of hardening.

As noted earlier, organomineral additives introduced into the cement system in the form of compositions (mixtures) with a superplasticizer with an optimized particle size distribution of aggregates make it possible to obtain high-tech self-compacting concretes. At the same time, such concrete mixtures are characterized by sufficient viscosity to prevent mixture separation and the sedimentation of large aggregates [10,36,37,38].

**Figure 12:**
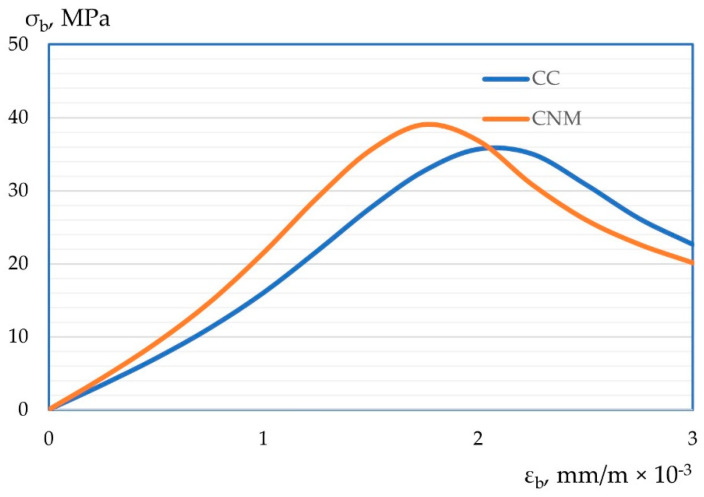
Stress-strain diagram for compression of self-compacting concrete.

**Figure 13:**
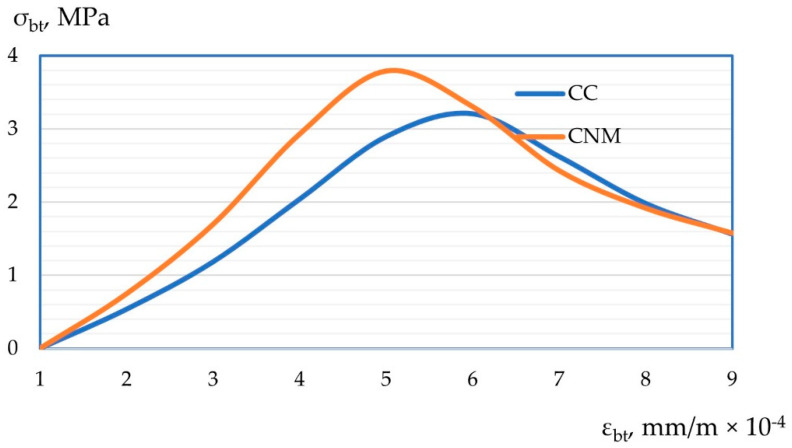
Tensile stress-strain diagram for self-compacting concrete.

Also, according to the test results, “stress-strain” diagrams of compression “ε_b_-σ_b_” and tension “ε_bt_-σ_bt_” were constructed. Graphic dependencies are shown in Figure 12 and Figure 13.

After experimentally obtaining “stress-strain” diagrams for cases of axial compression and cases of axial tension and their changes depending on the composition, their more detailed interpretation should be given with an explanation of the results obtained. So, the resulting stress-strain diagrams, both in compression and tension are presented in Figure 12 and Figure 13. Thus, the peak of the “stress-strain” diagrams with the most effective compositions, previously established according to the strength characteristics of concrete, shifts up and to the left relative to control samples made by a more traditional method. This confirms the theoretical and practical results of improving the mechanical and reducing the strain properties of nano-modified concrete obtained according to optimal parameters, both prescription and technological. The shift of the peak of both diagrams up and to the left refers to an increase in strength characteristics and better resistance of such concrete to strain, thereby confirming the hypothesis on the compaction of the concrete package with the help of nano-modifiers, a more structured arrangement of both macroparticles and aggregates of concrete, and microparticles, including dispersed nano-modifiers. Thus, we obtain concrete with an improved structure, which in turn is reflected in their stress-strain diagrams.

## 4. Discussion

A thorough evaluation of the data got in this research with the results of other authors should be made to assess the authors’ contribution to the science and practice of production and construction. To better assess, it is necessary to set the criteria for a comparative assessment, which can be expressed in three aspects. First, this is a research aspect. The second is the technological aspect. And the third aspect is the result got from the point of view of operation of the final product in real construction that are special structures with hard-to-reach concrete. Their evaluation is carried out according to various characteristics and requirements that apply to these concretes.

The structure of large-scale studies conducted by Professor Zaichenko N.M. [10,36,37,38] earlier, and associate professor Egorova E.V. [10] was taken as a template and pattern concerning a full-fledged comprehensive study of self-compacting concrete. In addition, the works of the authors were considered using various modifiers, which are raw finely dispersed components used as an additive to self-compacting concrete mixtures with their verification by the rheology of the mixtures and the performance properties of the concrete obtained from them [4,5,6,7,8,9,11,12,13,14,15,16,17,18,19,20,21,22,23,24,25,26,27,28,29,30,31,32,33,34,35,39,40,41,42,43,44,45].

Summarizing the results of the identified research differences, it can be noted that a large-scale study was conducted in this paper on the nano-modification of self-compacting concrete mixtures with additives of various types. The goal was to improve the technological characteristics of the mixture and the performance characteristics of the resulting concrete. The nano-modifiers were fly ash formed during the operation of the existing state district power station, and micro-silica, which is a waste of industrial production. As a result of the study, high technological and operational indicators of concrete mix and concrete were revealed. The ecological and economic efficiency of the proposed research method, with the proposed technology formulation, has been proven. Thus, the prospects of this study and further vectors of the development of this study have been determined.

From the point of view of the technological properties of the obtained self-compacting concrete mixtures, a quantitative assessment of our results is presented. The quantitative increase was up to 83% in the technological and rheological characteristics of the self-compacting concrete obtained.

The third aspect of comparison is the result demonstrated by the concrete obtained on the technological mixtures proposed in terms of operational characteristics. The quantitative increase in the strength characteristics was up to 19% and up to 22% for water resistance. The reduction in ultimate strains was up to 14% and the elastic modulus increased by 11%.

The increase in strength characteristics is in good agreement with the results of the authors, who previously investigated the use of complex modifiers in self-compacting concrete [10,13,20,21,23,24,31,32,33]. However, the use of a complex modifier earlier in the form of blast-furnace granulated slag and fly ash [13] or limestone [20] improved the rheological properties but reduced the strength characteristics [13] or slightly increased them [20]. Also, the addition of only fly ash does not allow obtaining high increases in the characteristics of self-compacting concrete [16,17]. At the same time, the effective dosage of fly ash obtained in this research is comparable with the dosages of ash previously used by other authors, which provided the best results (30–50%) [16,17,23,24,33,40].

Summarizing all three aspects, several elements of novelty can be noted. First, this research is based on large-scale studies of our colleagues around the world, considering the experience of previous studies. Second, the theory of nano-modification of self-compacting concrete mixtures was continued and developed, and specific practical results were obtained, which are proposed for development. The vector of this development is bringing nano-modifiers to an even more dispersed state. In this case, an assessment is made in terms of raw materials and the possibility of establishing the effect of even greater grinding of these components. This makes it possible to identify opportunities for further improving the manufacturability of mixtures and improving the performance characteristics of concrete. The engineering aspect of our study is proposed for implementation in those construction organizations and enterprises to produce commercial self-compacting concrete. These enterprises have the highest ecological burden on the environment and access a large array of accumulated industrial waste. Thus, these enterprises are ready to provide initial raw materials that have already been verified in studies as suitable for use.

The observed results can be explained by the fact that the precipitation rate decreases in sodium hydroxide solution, which indicates a finer granulometric composition of the resulting dispersion. In a highly alkaline medium, along with the dispersion of micro-silica aggregates, its dissolution also occurs. In the presence of alkali, silicon dioxide goes into solution in the form of a silicate ion, followed by interaction with water to form soluble monosilicic acid. When the amount of alkali is small, monosilicic acid can polymerize to form stable colloidal particles. In the process of formation of soluble sodium silicate, an additional dispersion of amorphous silica occurs with a transition to a highly concentrated state-a lyosol nanodispersed system. When micro-silica is dispersed in the medium of sodium hydroxide and superplasticizer solutions, a colloidal system stabilized by a surfactant is formed, which retains sedimentation stability for more than 10 days. When replacing 40% of Portland cement with fly ash, a positive effect of a complex additive in the form of a superplasticizer C-3 + NaOH (P4) is observed in terms of SCC resistance to delamination [10,36,37,38].

Discussing the obtained results and comparing our conclusions with previous literature studies [10,13,20,21,23,24,31,32,33,36,37,38], the high degree of efficiency of our proposals can be noted. The complex nano-modifier, including such wastes as micro-silica and fly ash, presented positive results and demonstrated a synergistic effect in terms of saving raw materials and environmentally friendly disposal. This allows for recommending the proposed method for practical implementation in the construction industry and for monolithic concreting of complex densely reinforced structures.

**Figure 14:**
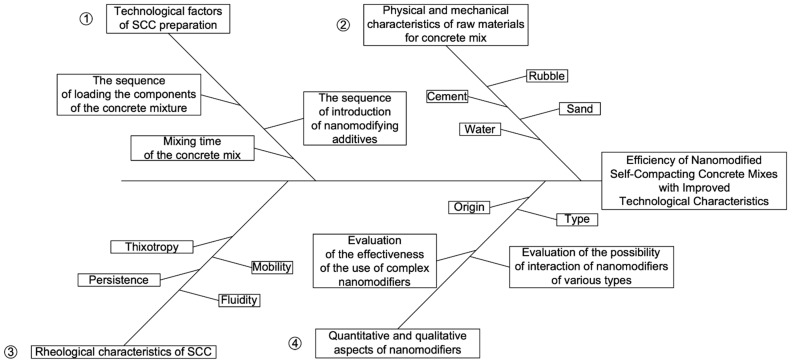
Causal diagram of obtaining effective nano-modified self-compacting concrete mixes with improved technological characteristics.

For a visualization of the analysis of the proposed solutions, Figure 14 shows a cause-and-effect diagram of the problem of obtaining effective nano-modified self-compacting concrete mixed with improved technological characteristics.

Thus, the most significant factors affecting the effectiveness of nano-modified self-compacting concrete mixtures with improved technological characteristics were grouped and analyzed.

Branch 1 (Figure 14) of the upper part of the diagram reflects the technological factors in preparing SCC mixtures that affect the quality of SCC mixtures as a final product. Branch 2 of the upper part of the diagram shows the physical and mechanical characteristics of the raw materials for the preparation of the concrete mixture, which also significantly affect the values of the final physical, mechanical and technological characteristics of the SCC mixture and the hardened SCC.

Branch 3 of the lower part of the diagram shows the rheological characteristics of SCC mixtures, the values of which directly affect the quality, scope and manufacturability of nano-modified SCC mixtures and composites based on them. On branch 4 of the scheme, the factor of influence of nano-modifiers is presented in quantitative and qualitative aspects, considering their type, origin, and joint interaction. The presented four branches play a decisive role in obtaining effective self-compacting concrete mixtures with improved technological characteristics and concrete based on them.

## 5. Conclusions

In conclusion, we theoretically and experimentally proved and substantiated the possibility of nano-modification using a complex modifier based on the industrial waste of self-compacting concrete and the possibility of improving the technological properties of concrete mixes using such nano-modifiers.

Experimental technological and mathematical dependencies between input factors—the characteristics of the technological process and raw materials and output parameters of the characteristics of concrete mixes and concretes—are determined. The optimization of these parameters was carried out, a theoretical substantiation of the obtained results has been proposed, and a quantitative picture has been presented, expressed in the increment of the properties of self-compacting concrete mixtures using nano-modifiers from industrial waste and concretes based on them. In terms of the mixture, these increases were 12% for slump-flow and 83% for fluidity concerning the control composition, while in terms of concrete, these increases were up to 19% for strength indicators and 22% for water tightness to the control composition. The ultimate strain was decreased by 14%, and elastic modulus increased by 11%.

A complex nano-modifier, including such waste as micro-silica and fly ash, gave positive results and demonstrated a synergistic effect in terms of saving raw materials and environmentally friendly disposal. This allows for recommending the proposed method for practical construction industry enterprises, as well as for monolithic concreting of complex densely reinforced structures.

An assessment of the results obtained is given and proposals for implementation in the practical industry are developed. According to preliminary estimates, the use of this nano-modifier leads to improving in the environmental situation because of the use of a nano-modifier based on industrial waste in concrete and results in a reduction of the process cost. The cost of the final product was decreased by reducing the cost of raw materials by up to 15%.

## Figures and Tables

**Table 1:** Chemical composition of Portland cement clinker.

Cement Type	Oxide Content, %											L.O.I.	Cl
	SiO_2_	Al_2_O_3_	CaO	Fe2O_3_	MgO	TiO_2_	P_2_O_5_	SO_3_	Na_2_O	K_2_O	Na_2_O_equiv._		
PC 500-D0-N	20.5	4.1	64.9	3.5	1.1	0.2	0.2	3.1	0.2	0.8	0.7	0.9	0.01

**Table 2:** Mineralogical composition of clinker.

Cement Type	Mineral Content, %				
	C_3_S	C_2_S	C_3_A	C_4_AF	CaO_fr._
PC 500-D0-N	74.0	8.6	4.0	11.8	0.7

**Table 3:** Physical and mechanical properties of Portland cement PC 500-D0-N.

Density, kg/m^3^	Blaine Specific Surface Area, cm^2^/g	Normal Density, %	Setting Time, min		Compressive Strength 28 Days, MPa	Bending Strength 28 Days, MPa
			Start	End		
3100	3512	26.4	160	212	59.2	6.9

**Table 4:** Chemical composition and physical and mechanical characteristics of fly ash from Novocherkassk GRES.

Indicators	Value
SiO_2_, %	45.92
TiO_2_, %	0.87
Al_2_O_3_, %	25.9
Fe_2_O_3_, %	9.38
CaO, %	0.81
MgO, %	1.35
MnO, %	0.36
K_2_O, %	5.29
Na_2_O, %	0.93
SO_3_, %	1.28
P_2_O_5_, %	0.15
L.O.I., %	7.37
Bulk density, kg/m^3^	480
True density, kg/m^3^	2600

**Table 5:** Chemical composition of micro-silica MS-85.

Material	Oxide Content, %							
	SiO_2_	Al_2_O_3_	Fe_2_O_3_	CaO	MgO	R_2_O	SO_3_	L.O.I.
MS-85	82.3	1.7	3.0	1.1	0.2	0.8	3.5	7.4

**Table 6:** Physical and mechanical properties of aggregates.

Material Title	Fineness Modulus *M*_f_ or Grain Sizes	Bulk Density, kg/m^3^	True Density, kg/m^3^	Void, %	Crushability, %
Medium quartz sand	2.2	1426	2649	46.8	-
Very fine quartz sand	1.2	1413	2653	44.2	-
Granite crushed stone	fraction 5–10 mm	1368	2670	45.6	11.8

**Table 7:** Qualitative characteristics of superplasticizer C-3.

Indicator Title	Value
Visual form	Homogeneous dark brown liquid
Density at 20 °C, no less, g/cm^3^	1.17
Water, wt. % no more	68.0
Hydrogen ion activity index (pH), 2.5% aqueous solution	8.0 ± 1.0

**Table 8:** Qualitative characteristics of NaOH.

Indicator Title	Value
Main substance, %	99.487
Sodium carbonate (Na_2_CO_3_), %	0.5
Sodium chloride (NaCl), %	0.005
Sodium sulfate (Na_2_SO_4_), %	0.005
Iron in terms of Fe_2_O_3_, %	0.002
Mercury content (Hg), %	0.0001

**Table 9:** Composition and properties of cement paste with a complex nano-modifier.

Denotation	Component Content, g					W/W	Ultimate Shear Stress, τ_0_, Pa
	PC	Fly-ash	MS-85	C-3	NaOH		
C1	500	-	-	-	-	0.25	59.7 ± 3.4
P1	500	-	-	5	-	0.20	56.7 ± 3.0
P2	-	500	-	-	1.5	0.29	56.0 ± 2.9
P3	300	200	-	-	1.5	0.26	57.6 ± 3.1
P4	300	200	-	5	1.5	0.22	58.1 ± 3.3
P5	300	200	24	5	1.5	0.24	58.0 ± 3.2

**Table 10:** Parameters of the composition of the concrete mixture.

Indicator Title	W/C	C, kg/m^3^	W, l/m^3^	CS, kg/m^3^	S, kg/m^3^	C-3, kg
Value	0.64	447	286	812	864	6.7

**Table 11:** Levels of input factors and intervals of their variation for 2nd order planning.

Input Factors		Level of Variation					Variation Interval δ
Factor Code	The Physical Meaning of the Factor	−2	−1	0	+1	+2	
X_1_	NaOH concentration by weight of cement (N), %	0.2	0.4	0.8	1.2	1.4	0.4 and 0.2
X_2_	Fly ash content (instead of part of cement) (F), %	25	30	40	50	55	10 and 5
X_3_	C-3 content by weight of cement (C), %	1.1	1.2	1.5	1.7	1.8	0.2 and 0.1
X_4_	The content of MS-85 by weight of cement (M), %	7	8	10	12	13	2 and 1

**Table 12:** Experiment design matrix.

Num	Variables				Natural			
	X_1_	X_2_	X_3_	X_4_	N	F	C	M
1	−1	−1	−1	−1	0.4	30	1.2	8
2	1	−1	−1	−1	1.2	30	1.2	8
3	−1	1	−1	−1	0.4	50	1.2	8
4	1	1	−1	−1	1.2	50	1.2	8
5	−1	−1	1	−1	0.4	30	1.7	8
6	1	−1	1	−1	1.2	30	1.7	8
7	−1	1	1	−1	0.4	50	1.7	8
8	1	1	1	−1	1.2	50	1.7	8
9	−1	−1	−1	1	0.4	30	1.2	12
10	1	−1	−1	1	1.2	30	1.2	12
11	−1	1	−1	1	0.4	50	1.2	12
12	1	1	−1	1	1.2	50	1.2	12
13	−1	−1	1	1	0.4	30	1.7	12
14	1	−1	1	1	1.2	30	1.7	12
15	−1	1	1	1	0.4	50	1.7	12
16	1	1	1	1	1.2	50	1.7	12
17	−2	0	0	0	0.2	40	1.5	10
18	2	0	0	0	1.4	40	1.5	10
19	0	−2	0	0	0.8	25	1.5	10
20	0	2	0	0	0.8	55	1.5	10
21	0	0	−2	0	0.8	40	1.1	10
22	0	0	2	0	0.8	40	1.8	10
23	0	0	0	−2	0.8	40	1.5	7
24	0	0	0	2	0.8	40	1.5	13
25	0	0	0	0	0.8	40	1.5	10
26	0	0	0	0	0.8	40	1.5	10
27	0	0	0	0	0.8	40	1.5	10
28	0	0	0	0	0.8	40	1.5	10
29	0	0	0	0	0.8	40	1.5	10
30	0	0	0	0	0.8	40	1.5	10
31	0	0	0	0	0.8	40	1.5	10

**Table 13:** Properties of concrete mixture and concretes.

Num	Yield, s	Slump-Flow, mm	Compressive Strength, MPa
1	14	500	46.9
2	15	440	39.2
3	15	480	32.5
4	16	450	29.7
5	15	610	49.7
6	16	630	48.5
7	17	600	30.8
8	17	620	31.7
9	13	490	47.9
10	12	460	37.5
11	15	450	33.6
12	16	440	28.7
13	26	520	48.4
14	25	610	45.9
15	21	600	31.3
16	26	610	28.9
17	23	670	40.7
18	24	460	35.8
19	24	460	52.8
20	26	450	27.8
21	13	410	45.8
22	26	640	47.9
23	14	460	44.9
24	15	450	44.9
25	23	540	46.3
26	21	550	45.8
27	22	550	46.8
28	20	540	45.5
29	24	540	44.9
30	23	540	47.9
31	25	550	46.9
Control	11	500	40.4

**Table 14:** Composition of self-compacting concrete mixes.

Mix	W/B	C, kg/m^3^	CS, kg/m^3^	S, kg/m^3^	C-3, kg	NaOH, kg/m^3^	MS-85, kg/m^3^	Fly-Ash, kg/m^3^
CC	0.48	447	812	864	6.7	-	-	-
CNM	0.48	268	812	864	4.8	2.14	26.8	179

**Table 15:** Rheological and physical-mechanical properties of self-compacting concrete with a complex nano-modifier.

Indicator Title	Value	
	CC	CNM
Slump-flow, mm	500	560
Fluidity, s	12	22
Compressive strength at the age of 2 days, MPa	21.9	25.9
Compressive strength at the age of 7 days, MPa	33.8	38.7
Compressive strength at the age of 28 days, MPa	46.9	51.9
Axial compressive strength at the age of 28 days, MPa	35.7	38.9
Axial tensile strength at the age of 28 days, MPa	3.2	3.8
Tensile strength in bending at the age of 28 days, MPa	5.6	6.2
Ultimate strains under axial compression, mm/m × 10^−3^	2.17	1.86
Ultimate strains in axial tension, mm/m × 10^−4^	1.26	1.09
Modulus of elasticity, GPa	31.4	34.8
Water resistance, MPa	1.25	1.53

## Data Availability

The study did not report any data.
